# Clinical Significance of Plasma Leptin and Its Receptors mRNA Expression in Craniopharyngiomas: A Prospective Study

**DOI:** 10.3390/biom13071078

**Published:** 2023-07-05

**Authors:** Youchao Xiao, Wentao Wu, Kefan Cai, Lu Jin, Yanfei Jia, Ning Qiao, Fangzheng Liu, Siming Ru, Lei Cao, Songbai Gui

**Affiliations:** Department of Neurosurgery, Beijing Tiantan Hospital, Capital Medical University, Beijing 100070, China

**Keywords:** craniopharyngioma, leptin, leptin resistance, obesity, progression-free survival

## Abstract

Craniopharyngioma (CP) is a benign tumor with a high rate of obesity and frequent recurrence. Moreover, the role of leptin/leptin receptors axis in obesity and the prognosis of CP is still unknown. Plasma leptin concentration and mRNA expression of leptin receptors were assessed in patients with CP. Moreover, the association between leptin/leptin receptors axis, weight-related outcomes, and progression-free survival (PFS) were explored in CP patients. Leptin receptors overexpressed in CP tumor tissue were compared to normal brain tissue (*p* < 0.05); compared to healthy controls, the concentration of leptin was elevated in CP with or without matched age, sex, and body mass index (BMI) (*p* < 0.05). The high plasma leptin level was an independent risk predictor for significant weight gain (adjusted odds ratio (aOR) = 2.29, and *p* = 0.030) and new-onset obesity (aOR = 6.64, and *p* = 0.016). High plasma leptin level (adjusted hazard ratio (aHR) = 3.74, and *p* = 0.011) and leptin receptor (LEPR) mRNA expression (aHR = 3.12, and *p* = 0.045) were independent risk factors for poor PFS in CP. Inappropriately elevated leptin relative to BMI and its failure to inhibit further weight gain indicate the existence of leptin resistance in patients with CP. Leptin and LEPR were independent predictors for PFS of patients with CP. The leptin/leptin receptors axis may be a potential therapeutic target for obesity in patients with CP.

## 1. Introduction

Craniopharyngioma (CP) is a rare brain tumor originating from the remnants of the craniopharyngeal duct epithelium and accounts for 2–5% of all primary intracranial tumors with an incidence equal 0.5–2.5 new cases per 1 million population annually [1]. Histopathologically, CP is considered benign (WHO grade I) and divided into two subtypes: adamantinomatous craniopharyngioma (ACP) and papillary craniopharyngioma (PCP). However, managing CP remains a unique challenge due to frequent recurrences and significant treatment-related morbidity [2]. Most notably, CP has been found to have a high prevalence of obesity (up to 50%) and obesity-related morbidity and mortality [3,4].

The hypothalamus is the center for regulating energy homeostasis [5], and the imbalance between energy intake, basal metabolism, and energy expenditure leads to obesity [6]. The hypothalamus nuclei sense a variety of peptides, such as leptin, insulin, and peptide YY, and nutrients, such as free fatty acids, glucose, and amino acids, to maintain energy homeostasis [7]. Hence, the damage of hypothalamic nuclei caused by the mass effect of tumors or subsequent treatments was strictly associated with hyperphagia and the high rate of obesity in patients with CP [8]. In addition, hypothalamic resistance to circulating peptides, such as leptin resistance, was also thought to contribute to the development of morbid obesity [9].

Leptin is a 16 kDa polypeptide that is mainly secreted from white adipose tissue, and it binds to the leptin receptor (LEPR), leptin receptor overlapping transcript (LEPROT), and leptin receptor overlapping transcript-like 1 (LEPROTL1) of hypothalamus neuro to increase metabolism and decrease food intake [10]. In patients with child-onset CP, the plasma leptin level is correlated with the presurgery body mass index (BMI) [11,12], while its associations with postsurgery BMI, weight change, and other clinical features are still not analyzed. Moreover, there is no consistency in whether patients with CP possess leptin resistance characterized by elevated leptin levels inappropriate for the degree of obesity. Furthermore, emerging evidence have proven that elevated expression of leptin and its receptors might potentially contribute to the progression of many tumors, including endometrioid endometrial cancer [10], breast cancer [13], and liver cancer [14]. Until now, there has been a lack of systematic studies focused on the association between leptin/leptin receptors and the progression of patients with CP.

For the past decades, managing obesity in CP patients was mostly focused on the hypothalamus sparing to prevent morbid obesity caused by hypothalamus damage, while the inherent hypothalamic insensitivity to peripheral metabolic signals has not yet been fully explored clinically. Therefore, this study aimed to determine the expression profile of plasma leptin and its receptors in tumor tissue samples of patients with CP, explore the association between the leptin/leptin receptors axis and weight change, new-onset obesity, and confirm the prognostic value of leptin and its receptors in patients with CP. We hope that these clinical discoveries provide insight into designing novel treatment strategies for preventing obesity in CP patients.

## 2. Materials and Methods

### 2.1. Patient Selection

**Figure 1:**
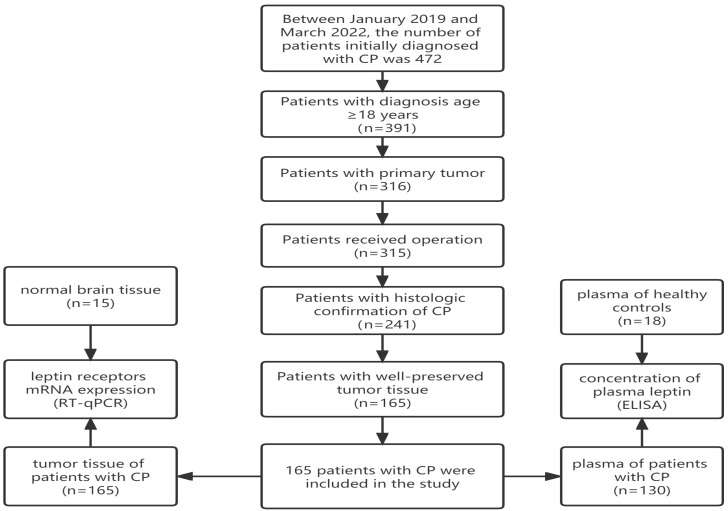
The flowchart of patient selection.

This study was approved by the Hospital Ethics Committee (Beijing Tiantan Hospital), and written informed consent was obtained from all patients or their legal guardians. We conducted a single-center cohort study of patients with CP who underwent endoscopic endonasal transsphenoidal surgery between January 2019 and March 2022. Criteria for patient selection were listed as follows: (I) diagnosis age ≥ 18 years; (II)patient was primary and did not receive irradiation or cyst aspiration before surgery; (III) histological diagnosis (ACP or PCP) was available and confirmed; (IV) patients with well preserved and test available tumor tissue; (V) patients were followed for at least 1 year. Finally, 165 patients met all the abovementioned criteria and were included in the study. The diagram of patient selection is shown in Figure 1.

### 2.2. Data Collection and Definition

Demographic characteristics (e.g., age, sex, and BMI), tumor features (e.g., tumor size, tumor type (cystic or not), and hypothalamic involvement (HI)), and relevant follow-up data (e.g., length of follow-up, recurrence, and postsurgery BMI) were collected for the analysis. Preoperative magnetic resonance imaging (MRI) was used to identify the maximum diameter of the tumor in three dimensions; tumor volume and cyst volume were calculated using the standardized method: volume = 4/3×π×(a/2×b/2×c/2), where the longest diameter in each plane measured variables [15]. Cystic tumors are those with more than 50% cystic tumor volume [15]. Three-dimensional computed tomography was completed preoperatively to confirm the presence of calcification. Regarding HI, Puget’s grading system was used to assess HI, which ranges from grade 0 to grade 2 [16]. Postoperative MRI was used to determine the extent of resection, including gross total resection (GTR, 100% removal), subtotal resection (STR, ≥95% removal), and partial resection (PR, <95% removal) [17]. In the statistical analysis, the latter two were combined into the non-gross total resection (NTR) group.

Patients were followed for postoperative BMI from primary surgery until the date of progression/recurrence, receiving radiotherapy, death, or last follow-up (1 March 2023), whichever came first [15]. In this study, progression-free survival (PFS) was defined as the time from operation to progression/recurrence or death, and tumor progression/recurrence was defined as the appearance of new lesions on MRI or the enlargement of residual tumors on follow-up [18]. For adults, we used standard WHO criteria to define overweight (BMI ≥ 25 kg/m^2^) and obesity (BMI ≥ 30 kg/m^2^). Consistent with one of our previous studies, postoperative BMI changes ≥5% were considered significant weight changes [15].

### 2.3. Real-Time Quantitative PCR (RT-qPCR)

Steadypure universal RNA extraction kit (Accurate Biotechnology, Hunan, China, Cat No. AG21017) was employed to extract total RNA. The concentration and quality of the obtained extracts were assessed via Nanodrop^®^ 2000 spectrophotometer (Thermo Fisher Scientific, Waltham, MA, USA). The value of the absorbance ratio 260/280 nm and 260/230 nm in the range of 1.9–2.1 allowed for reverse transcription. Subsequently, an Reverse Transcription kit (Biosharp Biotechnology, Anhui, China, BL696A) was utilized for reverse transcription. Then, synthesized cDNA was mixed with target primers and SYBR green qPCR Mix (Biosharp Biotechnology, BL698A) to amplify the target gene, and the amplicons were measured using QuantStudio 5 RealTime PCR System and Analysis Software v1.5.1 (Applied Biosystems, Foster City, CA, USA). The glyceraldehyde-3-phosphate dehydrogenase gene (GAPDH) was used as the housekeeping gene for normalization. The 2^−∆∆CT^ method was used to determine the gene expression levels of CP tissue relative to normal brain tissue (10 pituitary and 5 hypothalami), and the gene expression was transformed using the log_2_ (X + 1) function. Each experiment was repeated at least three times. The primers of the target genes and GAPDH were synthesized by Accurate Biotechnology and were listed in Table 1.

### 2.4. Measurement of Preoperative Plasma Leptin Protein

Blood samples were collected the day before the surgery, and the blood samples were centrifuged at 3000 rpm for 2 min, where the blood components were separated. Blood components were maintained at −80 °C until leptin determination was carried out. The concentration of leptin protein in the plasma was determined using the Human Leptin Standard ABTS ELISA Development Kit (Peprotech, Inc., Cat No. 900-K90) following the manufacturer’s instructions. Finally, the preoperative plasma leptin concentration was analyzed in 130 out of 165 patients included in the present study.

### 2.5. Statistical Analysis

Statistical analysis was performed and visualized using SPSS 24 (SSPS, Inc., Chicago, IL, USA) and GraphPad Prism 9 (GraphPad Software, Inc., La Jolla, CA, USA). Student’s *t*-test and Mann–Whitney U-test were used to analyze the parametric and nonparametric variables between groups. Chi-square and Fisher’s exact tests were used to compare categorical variables. Pearson correlation analysis was used to study the correlation between plasma leptin level and its receptors’ mRNA expression. Univariate logistic regression analysis and multivariate logistic regression analysis were used to identify the association between levels of plasma leptin and its receptors mRNA expression weight-related outcomes, including significant weight gain and new-onset postsurgery obesity. Kaplan–Meier (K-M) curve and log-rank test were conducted for the crude association between leptin, its receptors, and PFS, and multivariate Cox regression analysis was used to validate the independent predicting value of leptin and its receptors for PFS in patients with CP. Clinical parameters, including age, sex, histopathologic subtype, calcification, HI, the extent of surgical resection, and preoperative BMI, were adjusted in both multivariate logistical and Cox analyses. A *p*-value < 0.05 was considered statistically significant.

## 3. Results

### 3.1. Patient Characteristics

The clinical characteristics of the patients are shown in Table 2. The present study included a total of 165 patients (73 male and 92 female) with median age equaled to 47 years (ranging from 19 to 74). The average length of follow-up was 21.66 ± 10.05 months. Concerning the histopathological tumor subtype, histological examination revealed ACP in 123 patients and PCP in 41 patients. Based on MRI and the presenting symptom, such as headache and vomiting, 28.5% of patients (*n* = 47) were diagnosed with preoperative hydrocephalus. Calcification occurred in 59.4% of patients (*n* = 98). According to Puget’s grade system, patients were categorized into grade 2 HI (*n* = 79, 47.9%), grade 1 HI (*n* = 59, 35.8%), and grade 0 HI (*n* = 27, 16.4%). Details of individual pituitary hormone deficiency at presentation in the present cohort are as follows: gonadotropic hormone deficiency (*n* = 47, 28.5%) and antidiuretic hormone deficiency (*n* = 42, 25.5%) were the two most common hormone deficiencies in patients with adult-onset CP, followed by thyroid-stimulating hormone deficiency (*n* = 28, 17.0%), growth hormone deficiency (*n* = 25, 15.2%) and adrenocorticotropic hormone deficiency (*n* = 20, 12.1%).

The median tumor size (maximum diameter of tumor) and average tumor volume were 3.1 cm and 12.71 ± 12.74 cm3, respectively. The median cyst size (maximum diameter of cyst) was 2.0 cm (range from 0 to 7.4 cm), and the average cyst volume was 5.21 ± 7.94 cm3. More than half of the tumors (*n* = 94, 57%) were cystic tumors. All patients received surgical resection via endoscopic endonasal transsphenoidal surgery, and gross total resection was achieved in 90.3% of patients (*n* = 149); during the follow-up, 10 patients received radiotherapy for the tumor relapse.

### 3.2. Expression Levels of Leptin and Its Receptors

**Figure 2:**
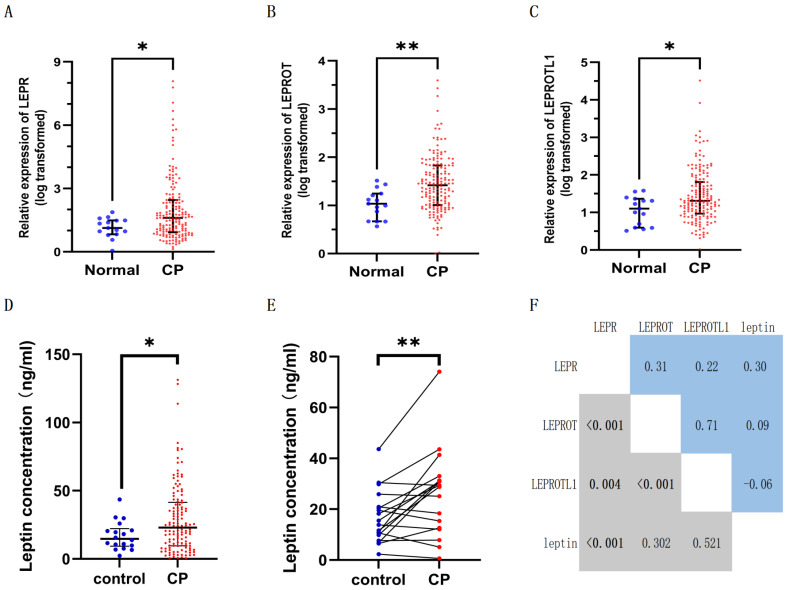
The mRNA expressions of (**A**) LEPR, (**B**) LEPROT, and (**C**) LEPROTL1 were elevated in the tumor tissue of CP compared to normal brain tissue. Compared to healthy controls, the plasma leptin concentration was higher in (**D**) patients with CP and (**E**) age, sex, and body mass index-matched patients with CP. (**F**) The correlation between plasma leptin and leptin receptors. LEPR, leptin receptor; LEPROT, leptin receptor overlapping transcript; LEPROTL1, leptin receptor overlapping transcript-like 1. * means *p* < 0.05, ** means *p* < 0.01.

As shown in Figure 2, the expression of leptin receptors, including LEPR (Figure 2A, 1.61 vs. 1.13, and *p* = 0.046), LEPROT (Figure 2B, 1.42 vs. 1.04, and *p* = 0.002), LEPROTL1 (Figure 2C, 1.31 vs.1.10, and *p* = 0.036), were elevated in CP tumor tissue compared to normal brain tissue. The median preoperative plasma level of leptin was 22.95 ng/mL (range from 0.5 ng/mL to 131.3 ng/mL). The concentration of preoperative plasma leptin was elevated in patients with CP compared to the control group (Figure 2D, 29.18 ± 25.56 vs. 16.90 ± 10.45, and *p* = 0.046; unpaired *t*-test); after matching age, gender, and BMI, the plasma leptin level was still significantly elevated than that of the control group (Figure 2E, 26.09 ± 17.10 vs. 16.90 ± 10.45, and *p* = 0.005; paired *t*-test). Furthermore, Figure 2F demonstrated that LEPR mRNA expression positively correlated with mRNA expressions of LEPROT (r = 0.31, and *p* < 0.001), LEPROTL1 (r = 0.22, and *p* = 0.004), and plasma leptin concentration (r = 0.30, and *p* < 0.001). In addition, the LEPROT mRNA expression also positively correlated with LEPROTL1 mRNA expression (r = 0.71, and *p* < 0.001).

### 3.3. Clinical Significance of Leptin and Its Receptors

Table 3 shows the association between clinical characteristics, leptin, and leptin receptors. LEPR was elevated in patients with ACP subtype (1.66 vs. 1.12, and *p* = 0.001), cystic tumor (1.65 vs. 1.21, and *p* = 0.001), and preoperative obesity (1.72 vs. 1.51, and *p* = 0.043). Elevated LEPROT was observed in patients with preoperative obesity (1.53 vs. 1.45, and *p* = 0.038), and LEPROTL1 was highly expressed in male patients with CP (1.38 vs. 1.20, and *p* = 0.031). In addition, the plasma level of leptin was associated with many traits of patients with CP, including higher-grade HI (25.85 vs. 16.95, and *p* = 0.037), preoperative obesity (29.55 vs. 21.13, and *p* = 0.035), and female gender (32.77 vs. 16.77, and *p* < 0.001). Moreover, the expression of LEPR positively correlated with the maximum diameters of tumor and cyst, as well as volumes of tumor and cyst (all r > 0.1 and all *p* <0.05). The result also showed that the plasma level of leptin also positively correlated with the size indexes, both tumor volume (r = 0.21, and *p* = 0.048) and maximum diameter of tumor (r = 0.16, and *p* = 0.045).

### 3.4. Association between Preoperative Plasma Leptin, Leptin Receptors, Weight Change, and New-Onset Obesity

There were 33 patients with obesity at the last follow-up, including 19 patients (3 patients with preoperative normal weight and 16 patients with preoperative overweight) who represented new-onset postoperative obesity during the follow-up and 14 patients with preoperative obesity (Table 4).

**Figure 3:**
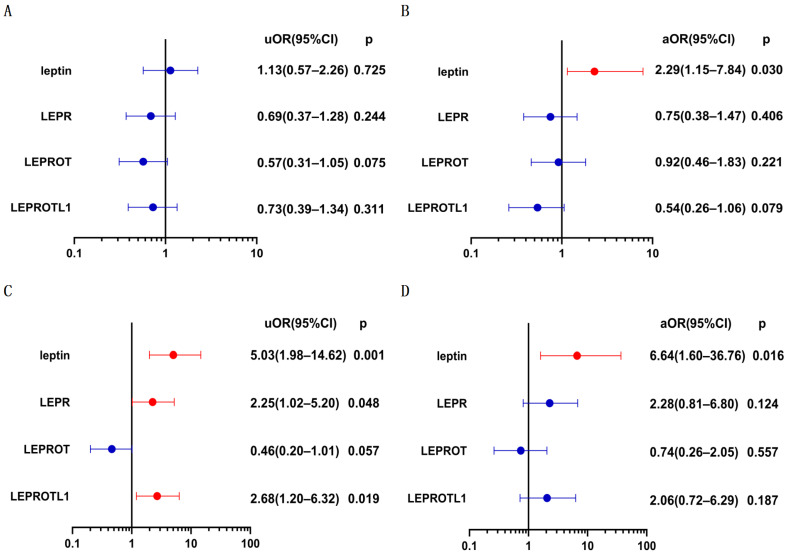
Univariate logistic analysis for (**A**) significant weight gain and (**C**) new-onset obesity. Multivariate logistic analysis for (**B**) significant weight gain and (**D**) new-onset obesity. Clinical parameters, including age, sex, histopathologic subtype, calcification, hypothalamus involvement, the extent of surgical resection, and preoperative body mass index, were adjusted in multivariate logistic analysis. uOR, unadjusted odds ratio; aOR, adjusted odds ratio.

The univariate logistical analysis showed that none of the factors were associated with significant weight gain, while the multivariate logistical analysis indicated that high preoperative plasma leptin level was a risk factor for significant weight gain (Figure 3B, adjusted odds ratio (aOR) = 2.29; 95% confidence interval (CI), 1.15–7.84; and *p* = 0.030). Concerning new-onset obesity, 15 patients with preexisting obesity were excluded from the logistical regression analysis, and the univariate logistical analysis showed that high preoperative plasma leptin level (unadjusted odds ratio (uOR) = 5.03; 95%CI, 1.98–14.62; and *p* = 0.001), high LEPR mRNA expression (uOR =2.25; 95%CI, 1.02–5.20; and *p* = 0.048), and high LERPOTL1 mRNA expression (uOR = 2.68; 95%CI = 1.20–6.32; and *p* = 0.019) were associated new-onset postoperative obesity; but the multivariate logistical analysis indicated that only high preoperative plasma leptin level was an independent risk factor for new-onset obesity (Figure 3D, aOR = 6.64; 95%CI, 1.60–36.76; and *p* = 0.016).

### 3.5. Prognostic Value of Leptin and Its Receptors

**Figure 4:**
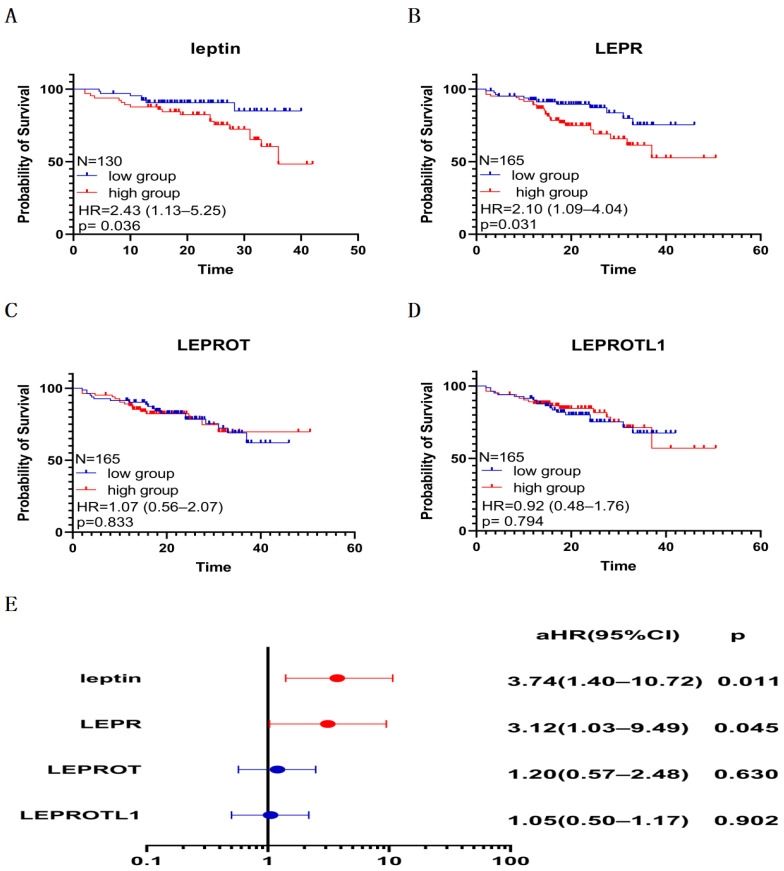
Prognostic value of plasma leptin and mRNA expression of leptin receptors. The Kaplan–Meier curves indicate that poor PFS was associated with (**A**) high plasma leptin and (**B**) LEPR mRNA expression but not (**C**) LEPROT mRNA expression and (**D**) LEPRROTL1 mRNA expression. (**E**) Multivariate Cox analysis shows that high plasma leptin and LEPR mRNA expression were independent risk factors for poor PFS in patients with CP. Clinical parameters, including age, sex, histopathologic subtype, calcification, hypothalamus involvement, the extent of surgical resection, and preoperative body mass index, were adjusted in multivariate Cox analysis. aHR, adjusted hazard ratio.

The K m curves showed a crude association between poor PFS and high plasma leptin level (Figure 4A, hazard ratio (HR) = 2.43; 95%CI, 1.13–5.25; and *p* = 0.036) and high LEPR mRNA expression (Figure 4B, HR = 2.10; 95%CI, 1.09–4.04; and *p* = 0.031). Furthermore, the multivariate Cox proportional hazards regression adjusting for confounding factors validated the independent prognosis value of plasma leptin (Figure 4E, adjusted HR (aHR) = 3.74; 95%CI, 1.40–10.72; and *p* = 0.011) and LEPR mRNA expression (Figure 4E, aHR =3.12; 95%CI, 1.03–9.49; and *p* = 0.045) for PFS in CP.

## 4. Discussion

CP is a benign and slow-growing tumor with overall survival (OS) ranging from 54 to 96% at 5 years, 40 to 100% at 10 years, and 62 to 85% at 20 years [1]. However, CP appears to have a high recurrence rate and high rate of cardiovascular mortality associated with morbid obesity, thus adversely affecting the quality of life of patients with CP. Hence, uncovering the pathogenesis and mechanism of morbid obesity and recurrence is challenging and is of great interest.

At a steady state, energy storage in adipose tissue equals food intake minus energy expenditure (storage = food intake—energy expenditure) [19], and the cumulative imbalance between energy intake and expenditure contributes to morbid obesity. The hypothalamus is the center for regulating blood pressure, heart rate, body temperature, as well energy balance via sensing peripheral metabolic signals, including leptin and insulin [20]. Therefore, the damage to the hypothalamus caused by tumors or treatments leads to overeating [21], decreased resting energy expenditures [22], and decreased physical activity [23,24], which further result in excess energy storage and morbid obesity in patients with CP [25].

Except for the disruption of the hypothalamic structure integrity, the existence of hypothalamic resistance to various circulation hormones was found to contribute to obesity, such as leptin [26,27]. Consistent with previous studies [23,28,29], we also found the existence of hyperleptinemia in patients with CP; compared to healthy controls, the mean concentration of plasma leptin was elevated in CP without or with matched age, sex, and BMI (Figure 2D, *p* < 0.05; Figure 2E, and *p* < 0.01). Furthermore, the multivariate logistic analysis showed that high leptin level was an independent predictor for significant weight gain (aOR = 2.29; 95%CI, 1.15–7.84; and *p* = 0.03) and new-onset obesity (aOR = 6.64; 95%CI, 1.60–36.76; and *p* = 0.016), which indicates that high plasma leptin fails to inhibit further weight gain in patients with CP. Considering the inappropriate elevated leptin levels relative to BMI and its failure to prevent further weight gain, the existence of leptin resistance in patients with CP may be a reasonable explanation. In other words, the patients with higher preoperative leptin levels may possess more severe leptin resistance and impaired leptin signaling, and thus, are more likely to experience further weight gain during follow-up.

So far, leptin has been identified as the most critical hormone in regulating energy balance [9], and multiple signaling cascades are initiated by its binding to long-form leptin receptors on the plasma membrane, including JAK-STAT signaling [30]. The leptin level was strictly associated with energy status. For example, insulin and leptin levels are increased in the energy-replete state, which decreases appetite and food intake; conversely, in the fasting state, leptin and insulin levels are decreased, subsequently initiating food intake [8,31]. The integration of leptin signaling pathways guarantees the role of leptin in patients with CP, while many factors in CP affect the energy-regulating role of leptin, including decreased receptors within the hypothalamus, low leptin binding activity [32], lack of sensitivity to endogenous leptin [23]. The elevated expression of inhibitors of leptin signaling, such as cytokine signaling 3, T cell protein tyrosine phosphatase, and protein tyrosine phosphatase 1B, results in insensitivity to leptin; the expression of inflammatory mediators is attributed to the expression of inhibitors via activation of kappa B kinase beta/nuclear factor kappa B (IKKβ/NF-κB) signaling [33]. Notably, a high level of inflammation mediators was observed in solid tissue and cyst fluid in patients with CP, and two independent studies reported that injection of cyst fluid into the cortex of rat or the hypothalamus of mice increased body weight [34,35]; thus, the inflammation mediators secreted from the tumor may play an essential role in leptin resistance and development of obesity, and more studies are needed to reveal the mechanisms.

Due to CP’s invasive nature and proximity to the surrounding structure, recurrence seems inevitable. In one of our previous studies, we identified Integrin α6 as the biomarker for the OS of patients with CP via bioinformatic analysis, but only limited patients (*n* = 21) were included in the survival analysis [36]. Several studies reported that leptin and leptin receptors might be involved in the development and progression of tumors, including colorectal cancer [37], breast cancer [38], and hepatic cancer [39]. Therefore, we attempted to validate the prognosis value of the leptin and leptin receptors in a larger cohort.

Leptin, unlike most hormones, does not trigger a negative feedback decrease in receptor expression but induces the expression of its receptors in hypothalamic neurons or cultured microglia [40,41,42]. This unique relationship between leptin and its receptors was also observed in tumor cells of CP; we found that plasma leptin level positively correlated with LEPR mRNA expression (Figure 2, r = 0.3, and *p* < 0.001), and we suspected that the unique regulation system is essential for signaling amplification and persistent effect in CP tumor cells. In addition, high LEPR mRNA expression and high plasma leptin levels were positively associated with the sizes and volumes of tumors and cysts (Table 3). Moreover, the patients with severe HI possess higher plasma leptin levels than those with no HI or mild HI (Table 3, 25.85 vs. 16.95, and *p* = 0.037). Furthermore, the K m curve showed that poor PFS was crudely associated with the high level of plasma leptin (Figure 4, HR = 2.43, and *p* = 0.036) and high LEPR mRNA expression (HR = 2.10, and *p* = 0.031), and the multivariate Cox analysis showed that levels of leptin and LEPR were independent risk factors for poor PFS in patients with CP. In mechanism, leptin’s interaction with its receptor also enables the activation of the MAPK-ERK, PI3K-AKT, and JAK-STAT pathways, which contributes to malignant traits of tumor cells, such as proliferation, transformation, survival, and self-renewal [13,30]. Coincidentally, the MAPK pathway activation is necessary for the pituitary gland to transform into PCP tumor-initiating cells [43]. Therefore, the leptin/leptin receptors axis may be involved in CP’s malignant traits and poor PFS via unique expression patterns and multiple pathways.

## 5. Conclusions

Leptin and its receptors were overexpressed in patients with CP, and high preoperative plasma leptin was independently associated with significant weight gain and new-onset obesity, indicating the potential role of leptin resistance in the pathogenesis of obesity in CP. Leptin and LEPR were independent risk factors for poor PFS in patients with CP. Therefore, therapeutic strategies based on leptin resistance and the leptin/leptin receptors axis offer options for solving morbid obesity in patients with CP in the future.

## Figures and Tables

**Table 1:** Gene-specific primers used in the RT-qPCR. LEPR, leptin receptor; LEPROT, leptin receptor overlapping transcript; LEPROTL1, leptin receptor overlapping transcript-like 1.

Gene	Primer Sequences	Length (bp)
LEPR	Forward primer:GTT CCG AAC CCC AAG AAT TG	20
	Reverse primer:AGA CCA CAG TTG TTG GCA TC	20
LEPROT	Forward primer:GCT TGG AGA GGC AGA TAA CG	20
	Reverse primer:AAT GTC CTG GGT CCA GAG TG	20
LEPROTL1	Forward primer:TGC AAT GTG GGA AGA AAT GA	20
	Reverse primer:AAG GAG GAA GCA GAG GAA GG	20
GAPDH	Forward primer:GCA CCG TCA AGG CTG AGA AC	20
	Reverse primer:TGG TGA AGA CGC CAG TGG A	19

**Table 2:** Clinical characteristics of patients with craniopharyngioma. ACP, adamantinomatous craniopharyngioma; PCP, papillary craniopharyngioma; GTR, gross total resection; NTR, non-gross total resection; SD, standard deviation; BMI, body mass index.

Clinical Characteristic.	Total Patients (*n* = 165)
Age (year, mean ± SD)	44.84 ± 13.02
(year, median, range)	47 (19–74)
Gender (male/female)	92/73
Follow up (month, mean ± SD)	21.66 ± 10.05
Pathology (ACP/PCP)	123/42
Calcification (yes/no)	97/68
Hypothalamic involvement (2/1/0)	79/59/27
Preoperative hydrocephalus (yes/no)	47/118
Extent of resection (GTR/NTR)	149/16
Radiotherapy (yes/no)	10/155
Maximum diameter of tumor (cm, median, range)	3.1 (1.2–7.8)
Tumor volume (cm^3^, mean ± SD)	12.71 ± 12.74
Maximum diameter of cyst (cm, median, range)	2.0 (0–7.4)
Cyst volume (cm^3^, mean ± SD)	5.21 ± 7.94
Cystic tumor (yes/no)	94/71
Preoperative BMI (kg/m^2^, mean ± SD)	
normal weight (*n* = 75)	22.34 ± 2.29
overweight (*n* = 75)	27.09 ± 1.29
obese (*n* = 15)	32.26 ± 1.74
Pre-operative endocrinopathy	
Thyroid-stimulating hormone deficiency (yes/no)	28/137
Adrenocorticotropic hormone deficiency (yes/no)	20/145
Gonadotropic hormone deficiency (yes/no)	47/118
Growth hormone deficiency (yes/no)	25/140
Antidiuretic hormone deficiency (yes/no)	42/123

**Table 3:** Association between plasma leptin, leptin receptors mRNA expression, and clinical characteristics.

Clinical Characteristic	LEPR Expression (*n* = 165)	P	LEPROT Expression (*n* = 165)	P	LEPROTL1 Expression (*n* = 165)	P	Leptin Concentration (*n* = 130)	P
Age		0.289		0.364		0.498		0.571
<60	1.60 (0.89–2.43)		1.41 (1.01–1.83)		1.31 (0.97–1.81)		24.30 (9.77–41.35)	
≥60	1.49 (0.87–1.85)		1.33 (0.75–1.69)		1.22 (0.91–1.63)		14.60 (8.27–48.30)	
Gender		0.439		0.063		**0.031**		**<0.001**
female	1.53 (0.90–2.73)		1.31 (0.97–1.64)		1.20 (0.89–1.69)		32.27 (18.05–55.05)	
male	1.59 (0.88–2.08)		1.47 (1.02–1.84)		1.38 (1.10–1.95)		16.77 (6.30–33.05)	
Pathology		**0.001**		0.880		0.198		0.702
PCP	1.12 (0.55–1.81)		1.49 (0.96–1.85)		1.48 (1.09–1.99)		21.52 (9.56–38.30)	
ACP	1.66 (1.00–2.56)		1.36 (1.03–1.78)		1.27 (0.92–1.71)		24.48 (10.11–51.46)	
Calcification		0.190		0.395		0.245		0.848
no	1.30 (0.79–2.05)		1.46 (1.06–1.83)		1.45 (1.01–1.87)		22.45 (7.33–49.63)	
yes	1.63 (0.92–2.45)		1.34 (0.97–1.78)		1.26 (0.92–1.74)		22.95 (10.06–37.29)	
HI		0.872		0.207		0.422		**0.037**
grade1 & grade 0	1.59 (0.85–2.41)		1.40 (0.95–1.76)		1.36 (0.89–1.97)		16.95 (6.43–28.52)	
grade 2	1.58 (0.95–2.32)		1.40 (1.07–1.83)		1.25 (1.00–1.71)		25.85 (11.06–45.54)	
Hydrocephalus		0.139		0.337		0.453		0.202
yes	1.60 (0.96–2.51)		1.40 (0.97–1.77)		1.30 (0.92–1.74)		25.93 (9.43–49.55)	
no	1.41 (0.78–1.85)		1.40 (1.06–1.86)		1.35 (1.01–2.05)		19.93 (9.77–35.35)	
Cystic tumor		**0.001**		0.756		0.453		0.77
no	1.21 (0.73–1.82)		1.44 (0.97–1.83)		1.29 (1.04–1.93)		22.57 (9.09–41.40)	
yes	1.65 (1.09–2.58)		1.38 (1.03–1.79)		1.31 (0.91–1.74)		22.95 (9.64–41.79)	
preoperative BMI		**0.043**		**0.038**		0.126		**0.035**
<30	1.51 (0.87–2.32)		1.45 (1.01–1.83)		1.31 (1.00–1.87)		21.13 (9.47–39.80)	
≥30	1.72 (1.06–2.73)		1.53 (1.15–1.97)		1.34 (0.98–1.95)		29.55 (15.19–46.18)	
	LEPR expression (*n* = 165)	P	LEPROT expression (*n* = 165)	P	LEPROTL1 expression (*n* = 165)	P	leptin concentration (*n* = 130)	P
Tumor size	0.12 (0.02 to 0.23)	**0.048**	0.08 (−0.24 to 0.47)	0.287	0.19 (−0.07 to 0.42)	0.224	0.21 (0.04 to 0.41)	**0.048**
Tumor volume	0.14 (0.03 to 0.28)	**0.032**	0.31 (0.12 to 0.53)	**0.042**	0.10 (−0.05 to 0.46)	0.203	0.16 (0.02 to 0.33)	**0.045**
Cyst size	0.22 (0.06 to 0.36)	**0.005**	0.03 (−0.13 to 0.18)	0.751	−0.10 (−0.25 to 0.06)	0.232	0.11 (−0.07 to 0.28)	0.233
Cyst volume	0.25 (0.10 to 0.39)	**0.001**	0.03 (−0.12 to 0.19)	0.664	−0.10 (−0.25 to 0.06)	0.232	0.11 (−0.06 to 0.29)	0.198

**Table 4:** Body mass index of patients at diagnosis and last follow-up.

Preoperative	Postoperative		
	Normal Weight (*n* = 49)	Overweight (*n* = 83)	Obesity (*n* = 33)
normal weight (*n* = 75)	40	32	3
overweight (*n* = 75)	9	50	16
obesity (*n* = 15)	0	1	14

## Data Availability

The data used in the current study are available from Youchao Xiao and Wentao Wu for reasonable use.
